# Association of daily screen time and sleep with concussion history on academic grades: a United States youth risk behavior survey study

**DOI:** 10.1016/j.pmedr.2026.103570

**Published:** 2026-07-13

**Authors:** Shawn R. Eagle, Natalie Sherry, David O. Okonkwo

**Affiliations:** Department of Neurological Surgery, University of Pittsburgh, Pittsburgh, PA, USA

**Keywords:** Concussion, Television, Sleep, Screens, Academics

## Abstract

**Objective:**

We evaluated the association between daily hours of sleep, daily hours of screen time, concussion history, and academic performance in a sample of adolescents.

**Study design:**

The United States Youth Risk Behavior Survey datasets from 2017 to 2021 were analyzed. The unweighted sample included 29,388 adolescents, representing an estimated 12,097,952 adolescents. We analyzed history within the last year of sport- or recreation-related concussion, daily television, video games, and computer usage (3+ hours/day on average), sleep (8+ hours of sleep/day), daily physical activity (60+ minutes/day on 5+ days/week), sport participation, and averaging “A” or “B” grades.

**Results:**

The moderation model (R^2^ = 0.10; *p* < 0.01) had significant main effects for sport- or recreation-related concussion (adjusted; aOR = 0.16), 3+ hours of screen time (aOR = 0.85), and 8+ hours of sleep (aOR = 1.92) on better academic performance. A significant interaction effect for sport- or recreation-related concussion and 3+ hours of screen time (aOR = 0.57, 95% CI: 0.47, 0.68) and 8+ hours of sleep (aOR = 1.99, 95% CI: 1.60–2.46) were also observed.

**Conclusion:**

Adolescents who reported a sport- or recreation-related concussion in the previous year and daily screen time 3+ hours per day or sleep <8 h per day had poorer academic performance than those who reported less screen time and reported more sleep.

## 1 Introduction

Concussion is a public health concern for United States (U·S) youth, with an estimated 3 million occurring annually. International consensus guidelines suggest behavioral modifications (e.g., optimizing sleep, minimizing screen time) help facilitate faster recovery (Patricios et al., 2023). Limited population-level data is available to support those recommendations, and reducing screen time has been the subject of some debate (Lima Santos et al., 2026; Macnow et al., 2022). In a small randomized controlled trial where adolescents were instructed to abstain from screens for the first 48 h post-concussion compared to patients with concussion with complete access to screens, a reduction of 4.5 days in recovery time was noted for the screen abstinent group (Macnow et al., 2021). However, patients were not blinded to their group allocation, and the intervention group still reported 130 min of screen time. Social desirability and recall bias were therefore significant concerns, as the authors noted (Macnow et al., 2021). One retrospective study of 712 children and adolescents found a small but statistically significant effect of screen time on higher symptom severity scores in a concussed group compared to an orthopedic injury group (Cairncross et al., 2022). The nonlinear effect revealed that those in the 25th and 50th percentiles of screen time use were less symptomatic than those on the tail end of the distribution. Moreover, female sex and post-acute napping were stronger predictors of higher post-injury symptoms while more self-reported sleep overall was associated with fewer post-injury symptoms.

Sleep quantity and quality have long been regarded as key variables to support a healthy recovery from concussion (Ludwig et al., 2020). Prior studies have suggested that poor post-injury sleep quality can prolong concussion recovery by an average of 2 weeks in adolescents (Chung et al., 2019). Adolescents who sleep less than normal and those who sleep more than normal after concussion tend to experience more symptoms (Leddy et al., 2023). In a retrospective study of children and adolescents seen at a concussion specialty clinic, sleeping <7 h per night was associated with longer average concussion recoveries (61 days) compared to those who slept 7–9 h (38 days) and 9+ hours (34 days) (Cassimatis et al., 2023). While prior studies have focused on concussion recovery, sleep and screen time are likely to interact with concussion to influence other aspects of adolescent life, like academic performance. To date, this type of study has not been conducted but could be impactful to clinical practice, as parents and adolescents report significant concerns with academic performance after concussion (DeMatteo et al., 2022; Ransom et al., 2015). In this analysis, we evaluated the association between daily hours of sleep, daily hours of screen time, concussion history, and academic performance in a national sample of United States adolescents.

## 2 Methods

### 2.1 Study design and population

The Youth Risk Behavior Survey is a biennial survey of adolescent health risk behaviors conducted by the Centers for Disease Control and Prevention (Brener, 2024). The Youth Risk Behavior Survey is a nationally representative sample of United States adolescents (ages 12–18 years) compiled with a 3-stage cluster sampling method. The datasets from 2017, 2019, and 2021 were combined for analysis. This study was exempt from institutional review board approval due to the use of deidentified, publicly available data.

### 2.2 Measures

The following psychosocial variables were extracted for analysis including: history within the last year of sport- or recreation-related concussion, daily television (3+ hours/day on average), daily time spent playing video or computer games or using a computer (3 + hours/day on average), feeling sad or hopeless within the last year, sleep (8+ hours of sleep/day), daily physical activity (60+ minutes/day on 5+ days/week), sport participation, and averaging “A” or “B” grades. These were coded as binary variables (yes/no) by YRBS and the dichotomization of these variables was pre-specified by YRBS. The screen time variable was operationally defined as a “yes” if the respondent reported 3 + hours/day on average for television watching or 3 + hours/day on average for video games/computer usage.

### 2.3 Statistical analysis

Two general linear binomial models evaluated the moderation of sleep and television on sport- or recreation-related concussion and academic performance controlling for age (as a continuous variable), gender, race/ethnicity, physical activity and sport participation. Post-hoc model diagnostics were conducted to ensure the models did not violate statistical assumptions. Weighted analyses were conducted using the “survey” package in R. Adjusted odds ratios (aOR) and 95% confidence intervals (CIs) are reported from the models. Statistical significance was *p* < 0.05. R (v. 4.5.2) software was used for all analyses.

## 3 Results

The unweighted sample size included 29,388 adolescents, representing an estimated 12,097,952 adolescents (Table 1). Ages included 12–18 years, with 49% females and 48% white respondents. Overall, 27% reported participating on at least 1 sports team and 30% reported 1+ sport- or recreation-related concussions in the last year. Only 18.5% of respondents reported sleep 8+ hours per day and 39% reported 3+ hours of screen time per day. Fifty-five percent of the sample reported “A” or “B” grades, on average.

**Table 1:** Descriptive statistics for the unweighted sample of adolescent respondents to the United States Youth Risk Behavior Survey from 2017 to 2021 (*n* = 29,388).

**Age**	
*12*	41 (0.10%)
*13*	37 (0.10%)
*14*	3892 (13.00%)
*15*	7209 (25.00%)
*16*	7624 (26.00%)
*17*	7245 (25.00%)
*18*	3340 (11.00%)
**Sex**	
*Male*	14,881 (51.00%)
*Female*	14,507 (49.00%)
**Race/Ethnicity**	
*American Indian/Alaskan Native*	254 (0.90%)
*Asian*	1490 (5.10%)
*Black/African American*	4532 (15.00%)
*Native Hawaiian/Pacific Islander*	160 (0.50%)
*Hispanic/Latino*	2736 (9.30%)
*Multiracial/Hispanic*	4500 (15.00%)
*Multiracial/Non-Hispanic*	1606 (5.5%)
*White*	14,110 (48.00%)
**1+ Concussions in the Last 12 Months**	8672 (30.00%)
**Participating in 1+ Sports Teams**	7869 (27.00%)
**Physical Activity 60+ Minutes per Day/**	
**5 Days per Week**	10,718 (36.00%)
**Sleeping 8+ Hours Per Day**	5427 (18.50%)
**Hours of Sleep Per Day**	
*4 or less*	15,761 (38.40%)
*5*	4158 (10.10%)
*6*	6814 (16.60%)
*7*	7781 (19.00%)
*8*	4926 (12.00%)
*9*	1204 (2.90%)
*10 or more*	372 (0.90%)
**Watching 3+ Hours of Television Per Day**	11,316 (39.00%)
**Hours of Television Per Day**	
*0*	13,522 (32.30%)
*<1*	7840 (18.70%)
*1*	5470 (13.00%)
*2*	6173 (14.70%)
*3*	3504 (8.30%)
*4*	1949 (4.60%)
*5 or more*	3568 (8.50%)
**“A” or “B” Grades on Average**	16,243 (55.00%)

The moderation model (R^2^ = 0.10; *p* < 0.01) had significant main effects for sport- or recreation-related concussions (aOR = 0.16), 3+ hours of screen time per day (aOR = 0.85), and 8+ hours of sleep per day (aOR = 1.92) on better academic performance. A significant interaction effect for sport- or recreation-related concussions and 3 + hours of daily screen time (aOR = 0.57, 95% CI: 0.47–0.68; Table 2) and 8+ hours of daily sleep (aOR = 1.99, 95% CI: 1.60–2.46) were also observed. Older age (aOR = 1.12), male gender (aOR = 0.65), participating in sports (aOR = 0.29), daily physical activity (aOR = 0.65), and American Indian/Alaskan Native (aOR = 0.59), Black (aOR = 0.52), Hispanic/Latino (aOR = 0.44), Multiracial-Hispanic (aOR = 0.62), and Multiracial Non-Hispanic (aOR = 0.81) were also significantly associated with better academic performance.

**Table 2:** General linear binomial model for the interaction of sport or recreation-related concussion and reporting screen time usage 3+ hours/day (top) and 8+ hours of sleep/day (bottom) on reporting “A” or “B” grades using Youth Risk Behavior Survey data from 2017 to 2021.

	Adjusted Odds Ratio	95% Confidence Interval
Age	1.12	1.08, 1.16
Gender	0.65	0.54, 0.77
Race/Ethnicity (ref: White)		
*Asian*	0.59	0.40, 0.87
*Black*	1.22	0.71, 2.11
*Native Hawaiian/Pacific Islander*	0.52	0.40, 0.66
*Hispanic/Latino*	0.74	0.46, 1.18
*Multiracial-Hispanic*	0.44	0.34, 0.58
*Multiracial-Non Hispanic*	0.62	0.49, 0.80
*American Indian/Alaskan Native*	0.81	0.66, 0.99
Feeling Sad or Hopeless	1.51	1.38, 1.65
Sport- or recreation-related concussion	0.16	0.13, 0.20
Screen Time 3+ Hours/Day	0.85	0.76, 0.95
8+ Hours of Sleep/Day	1.92	1.63, 2.25
Sport Participation	0.29	0.23, 0.37
Daily Physical Activity	0.65	0.54, 0.77
Sport- or recreation-related concussions × Screen Time 3+ Hours/Day	0.57	0.47, 0.68
Sport- or recreation-related concussions × 8 Hours of Sleep/Day	1.99	1.60, 2.46

## 4 Discussion

In this nationally representative, weighted sample of United States adolescents, the interaction of reporting at least 1 sport- or recreation-related concussion in the last year with screen time of 3+ hours per day was associated with lower odds of “A” or “B” grades compared to those who watched <3 h per day. Conversely, the interaction of reporting at least 1 sport- or recreation-related concussion in the last year with sleeping 8+ hours per day was associated with higher odds of “A” or “B” grades compared to respondents with sport- or recreation-related concussion who slept <8 h.

Healthy sleep is crucial for appropriate development in adolescents; the need for better sleep after a head injury is unsurprising. While sleep dysfunction is known to influence poor outcomes after sport- or recreation-related concussion (Sufrinko et al., 2019), there is limited guidance on the amount of daily sleep required after sport- or recreation-related concussion (Patricios et al., 2023; McCrory et al., 2017). Generally, the American Academy of Sleep Medicine and National Sleep Foundation recommend adolescents get 8–10 h of sleep per 24 h to promote general health (Hirshkowitz et al., 2015). However, both insomnia and hypersomnia (i.e., sleeping 10+ hours per night) increase odds of worse outcomes after sport- or recreation-related concussion (DuPrey et al., 2022). In one adolescent study, sleeping fewer than 7 h was associated with higher symptom scores but sleep >9 h was associated with worse cognitive performance in visual memory, visual motor speed and reaction time (Kostyun et al., 2015). The underlying biological mechanism linking hypersomnia and poorer recovery after sport- or recreation-related concussion is not well understood, but authors have postulated impairments to the glymphatic system or maintenance of circadian rhythm as potential targets (Piantino et al., 2022). In the present study, sleeping 8+ hours per day was associated with higher likelihood of “A” and “B” grades when also reporting an sport- or recreation-related concussion in the previous year, but caution for oversleeping (>9–10 h) after sport- or recreation-related concussion may be warranted based on prior studies.

Given the ubiquity of electronic screens in modern life and their integration in United States academics and workplaces, the amount of healthy screen time per day is difficult to determine. Higher levels of screen time could therefore reflect engagement with typical activities of daily living, such as computer use for homework and structured video game play. Engagement with television, video games, and computers may be a more cognitively active process than engaging with social media on a cell phone, for example, which may be associated with better recovery trajectories and better academic performance. Cell phone screen time was not included in the Youth Risk Behavior Survey dataset, which limits the interpretability of the current operational definition of screen time. More granular information about the timing of screen engagement in relation to injury and recovery could help delineate clinical guidelines for screen use after sport- or recreation-related concussion. The present study adds meaningful context to this clinical question by providing nuance that more screen time is not necessarily worse, at least based upon the operational definitions used.

Many potential mechanisms for why more screen time may be a risk factor for worse outcomes after sport- or recreation-related concussion, including disruption of circadian rhythm and reducing sleep quality (Cairncross et al., 2022). Engagement with screens can also provoke symptoms associated with sport- or recreation-related concussion, such as headache, dizziness, and ocular symptoms, which some authors have suggested may delay recovery (Macnow et al., 2021; Rabaza et al., 2023). However, modern sport- or recreation-related concussion management now stipulates that normal activities of daily living should be re-initiated after 24–48 h of relative rest. Avoiding screens due to symptom provocation can induce feelings of isolation and detachment, especially for adolescents who use cell phones as a primary mechanism for social interaction (Lima Santos et al., 2026). Exposing adolescents to screens, even if it provokes some symptoms, should be encouraged early in sport- or recreation-related concussion recovery with targeted periods of brief rest if symptoms become too severe. This “expose-recover” model can prevent prolonged avoidance and facilitate faster recovery (Durfee et al., 2026).

### 4.1 Limitations

The Youth Risk Behavior Survey is a self-report survey which may be subject to recall bias, especially considering that participants are asked to recall events over the last year. Binary variables were analyzed to maximize interpretability of the findings, but this could have reduced the amount of variance captured by the statistical models. The causal relationship between television, sleep and academic performance in adolescents with sport- or recreation-related concussion history cannot be inferred from the cross-sectional study design. Future work should investigate the importance of screen content and timing in relation to sleep on academic performance in adolescents with recent sport- or recreation-related concussion history. Key covariates which impact academic performance, such as socioeconomic status and parent education level, were not available in this dataset. The pseudo-R2 values presented here are low (10%), indicating the models explain little of the variance in academic performance. Missing key covariates like socioeconomic status and parent education could be related to the low variance accounted for by the models. The Youth Risk Behavior Survey dataset does not capture time spent on a mobile phone, which is a critical aspect of screen time for modern adolescents.

## 5 Conclusions

Adolescents who reported a sport- or recreation-related concussion in the previous year and daily screen time 3+ hours per day or sleep <8 h per day had poorer academic performance than those who reported less screen time and reported more sleep.

## Authors contribution

Dr. Shawn Eagle conceptualized and designed the study, conducted the analysis, drafted the initial manuscript, critically reviewed and revised the manuscript, and approved the final manuscript for submission. Drs Natalie Sherry and David Okonkwo conceptually designed the study, contributed to writing the methods and discussion sections, and critically reviewed and revised the manuscript and approved the final manuscript for submission. All authors approved the final manuscript as submitted and agree to be accountable for all aspects of the work.

## CRediT authorship contribution statement

**Shawn R. Eagle:** Writing – review & editing, Writing – original draft, Software, Methodology, Conceptualization. **Natalie Sherry:** Writing – review & editing, Methodology, Investigation. **David O. Okonkwo:** Writing – review & editing, Investigation, Data curation.

## Funding statement

No funding was secured for this study.

## Declaration of competing interest

The authors declare that they have no known competing financial interests or personal relationships that could have appeared to influence the work reported in this paper.
